# Revealed reality of cultivation and licit/illicit use of Cannabis (*Cannabis sativa* L.) in the western mid-hills of Nepal: a list experiment

**DOI:** 10.1186/s42238-025-00276-w

**Published:** 2025-04-12

**Authors:** Thomas Michael Kloepfer, Shinji Kaneko, Niraj Prakash Joshi

**Affiliations:** 1 TAOYAKA Program, Hiroshima University, Higashihiroshima, Japan; 2 Graduate School of Innovation and Practice for Smart Society, Hiroshima University, Higashihiroshima, Japan; 3 The IDEC Institute, Hiroshima University, Higashihiroshima, Japan; 4 Graduate School of Humanities and Social Sciences, Hiroshima University, Higashihiroshima, Japan; 5 Network for Education and Research on Peace and Sustainability (NERPS), Hiroshima University, Higashihiroshima, Japan

**Keywords:** Rural livelihood, List experiments, Cannabis, Item count technique

## Abstract

Cannabis has a long history of utilization in various regions around the world for different purposes. In western Nepal, Cannabis is specifically used as a food crop, textile material, and lucrative cash crop, although the Nepali Narcotic Drug Control Act of 1976 ultimately prohibits Nepali farmers from sowing, trading, or selling their Cannabis crop. Regardless of this act, farmers are continuing these practices illegally, especially in remote regions where Cannabis has grown for centuries. The purpose of this study was to understand the current social acceptability of Cannabis cultivation in rural Nepal and to consider the future potential for legitimate cultivation. We tested two interconnected behaviors associated with Cannabis farming in the hill regions of western Nepal. The item count technique was applied to estimate the number of farmers who actively sow or plant Cannabis, and may also be selling Cannabis husks, a byproduct used in illicit drug production, for income generation. We compared our item count treatment with a direct questionnaire for farmers within the same region. Our results indicate that planting Cannabis is not a sensitive behavior, as more than 97% of the respondents directly stated that they plant Cannabis. The item count technique, however, did indicate that selling Cannabis husks is a sensitive behavior, where 29% were shown to sell husks when they were asked using the item count technique, whereas 2% of the farmers confirmed selling husks when asked directly. We also found that all the households in our study consumed seeds, which are highly nutritious food crops, both at home and at community gatherings. Direct questionnaire surveys also revealed that farmers can trade their seed crops for goods such as salt, soap, and oil or sell them for cash income. Thus, the current law, which is based solely on drug control, needs to be changed on the basis of the revealed multifaceted values and realities, including health, culture and rural economic development.

**Supplementary Information:**

The online version contains supplementary material available at 10.1186/s42238-025-00276-w.

## Introduction

Cannabis (*Cannabis sativa* L., used Cannabis hereafter) is one of the oldest cultivated nonfood crops grown since the Neolithic period, originally for fiber (Damania 1998). In addition, it has gained importance for food and medicine throughout human history worldwide. The crop has two distinct names, hemp and marijuana (Shrestha et al. 2022), depending on its use. Hemp is used for medicinal purposes, fiber, and food, whereas marijuana is used for recreational use because of its intoxicating properties and medicinal value (Schulttenhofer & Yuan 2017). Nepal is a country dominated by Hindus, and the crop has enormous significance in the country because of its connection to Lord Shiva, one of three most revered gods, and it is used for self-purification and self-mastery. Hence, the cultivation of Cannabis has a long history in Nepal, imbedded in many agricultural practices, religious practices, and rural economic practices of the past and present (Gersony 2003; Clarke 2007; Fisher 2011; Clarke & Merlin 2013).

Traditionally, the crop has three main uses i.e., cultural, medicinal, and recreational (Bhattarai 1992; Sapkota 2008; Fisher 2011; Clarke & Merlin 2013; Bhatt et al. 2021; Shakya et al. 2021; Shrestha et al. 2022). First, it is used by *yogis* as an aid to meditation, and male devotees as a symbol of fellowship, especially in their frequent *bhajans*, singing a devotional song in a group; second, it has ayurvedic and ethnomedicinal value and hence is used for a wide variety of medicinal purposes (Bhattarai 1992; Sapkota 2008; Clarke & Merlin 2013; Bhatt et al. 2021; Shrestha et al. 2022); third, it is used recreationally, mainly by old people, who consider themselves too old to work in the field, consuming the crop occasionally for enjoyment, pleasure or passing time (Fisher 2011). Harvesting of food (seed/*bhaang*), fiber (stem), and medicine (seed and leaves) from a single Cannabis crop was reported by H. B. Hodgson, the British colonial officer, in 1855 as a unique Nepali tradition, which was not reported from other regions (Hodgson 1855). The culture of Cannabis production was described as ‘the most profitable of any’, as *churrus* (hashish) and *ganja* (marijuana) cover the expense of culture (Royle 1855). Shrestha et al. (2022) also reported multiple uses of Cannabis. This multifaceted use of Cannabis in Nepal draws only a thin line between Cannabis as hemp and marijuana. The intention of farmers is to consider the crop as either hemp or marijuana, leading to adjustments in cultural practices. For example, plants are closely spaced and harvested prior to flowering if they are grown for fiber, and they are widely spaced and harvested at maturity if they are grown for seeds. Farmers also prefer growing Cannabis spaced widely if they are targeting the resin for marijuana and hashish (Clarke 2007). The multifaceted use of Cannabis in Nepal has existed through centuries-old learning to accommodate, regulate, and restrict Cannabis use within traditional and secure limits (Gersony 2003; Fisher 2011).

The open availability of marijuana and hashish started attracting the attention of ‘hippie’ to Kathmandu, Nepal, in the mid- 1960 s. Consequently, the price of hashish and marijuana sharply increased along with illegal exports to India (Fisher 2011). The government of Nepal started regulating the burgeoning market through the promulgation of the Intoxicants Act (1961) and the Intoxicants Rules (1962). This legislation established a system of excise and sales taxes through licensing the buy/sell and commercial cultivation of Cannabis (hashish and marijuana). However, there was internal pressure caused by the common belief that the ‘hippie invasion’ influences local youth, as well as external pressure from the US government as part of its ‘war on drugs’, and the UN, as the International Narcotics Control Board, regarded Cannabis as a grave and insidious danger (Fisher 2011). These pressures led the government of Nepal to revoke all licenses to cultivate, buy and sell marijuana on July 16, 1973, which was further institutionalized through the Narcotics Drug Control Act (1976) (Fisher 2011). The act ultimately prohibits Nepali farmers from sowing cannabis; harvesting materials from plants; and ultimately consuming, trading, or selling their crops to sustain their livelihood (Gersony 2003). The immediate effects were significant. The government has lost revenues from cannabis trade and tourism, whereas farmers have lost their lucrative cash crop, and middlemen and retail traders have lost their livelihoods (Fisher 2011). The farmers perceived that the government had taken the food out of their children’s mouths by banning marijuana cultivation. Thus, the impacts on farmers in the Western hills were so grave that women had to liquidate their silver necklaces and gold jewelry, and men had to migrate in greater numbers for their families'survival (Gersony 2003).

Despite the law, farmers are continuing Cannabis cultivation illegally, both near urban areas and in remote isolated regions (Gersony 2003). Shah (1997) reported that local farmers are growing Cannabis to harvest seed for food. Even laws illegalizing Cannabis production, regardless of its use, are never enforced in extremely remote regions such as upper Darchula (Clarke 2007). Similarly, Bhatt (1977) reported that a large area of land in the central, western, and far-western regions is cultivated Cannabis. Even the farmers in Makwanpur from central Nepal have had to revert back to Cannabis cultivation to deal with the hardship caused during the COVID- 19 global pandemic resulting in low performance of conventional agriculture (Bista 2020). It is even evidenced by the routine destruction of Cannabis plants by Nepal police that Cannabis is grown and used in many parts of the country at present as well (Adhikari & Shiwakoti 2020; Aryal & Adhikari 2019; Bista 2020). Seed, hashish, and marijuana are extracted from many Cannabis-growing regions of Nepal. Fiber and fiber-based entrepreneurship is not restricted in western Nepal (Clarke 2007). However, despite the socioeconomic and religious-cultural significance of this crop, there is limited information and data available on Cannabis cultivation in Nepal. Hence, the crop is listed under ‘data deficit species of Nepal’ under the neglected and underutilized species having a prospect as a future smart food (Joshi, Shrestha, Gautam, Poudel, & Gotame, 2019). Under the context that the crop cultivation is illegal in Nepal, the research scope in exploring management practices for its efficient production is very limited. However, research on Cannabis production and consumption in Nepal will be crucial in paving the path towards its legalization, at least relaxing the blanket ban, thereby dealing with the problem of poverty and inefficient agricultural practices persistent in rural Nepal. As Bista (2020) reported, Cannabis remains an important crop for farmers in rural Nepal when the supply chain of agricultural inputs like chemical fertilizers for conventional farming is disrupted and can help them move-out of poverty caused by poor agriculture production due to the disruptions as the crop performs well even with low input use. It is also equally important to understand the status of its illicit use in order deal with the possible harm caused by its legalization. Despite reporting, such illicit use is hindered by the social desirability bias, we were unable to trace any academic papers considering the social desirability bias while studying the status of its illicit use.

With this background, this paper aims to explore Cannabis cultivation in rural Nepal in terms of the extent of cultivation and the selling of husks as a form of income for farmers. Under the current discussion of legalizing Cannabis cultivation in Nepal to realize its contribution to household economies in rural areas and thereby the national economy in several formal (including lower and upper households of parliament) and informal forums, this paper provides valuable insights for related policy formulation (Khanal et al. 2021; Pathak, et al. 2024). This paper particularly studies the cultivation status of Cannabis for its variety of uses by overcoming the social desirability bias because of its illegal status. This is more important in the context where countries such as Canada, Uruguay, Thailand, most states in America, the Australian Capital Territory in Australia, have recently legalized Cannabis cultivation and opened regulated markets for its medical and recreational use, with many other countries creating legalized usage (Pathak, et al. 2024). This offers a reemerging opportunity for Nepalese farmers with extensive experience growing this crop and the potential contribute to a thriving global market. Thus, a deep and thorough investigation of Cannabis utilization, the necessary regulatory policies, and social impact on society is needed more than ever before (Pardo 2014; Grucza, et al. 2018). Weiss et al. (2017) state that “science must be front and center in this important policy debate. Notably, the United Nations 1988 Convention Against Trafficking Narcotic Drugs and Psychotropic Substances reviewed *“measures to eradicate illicit cultivation of narcotic plants”.* Article 14.2 briefly mentions the following measures: *“fundamental human rights, respect for the traditions of national and regional groups, and the protection of the environment"* (The United Nations 1998).

The potential contribution of this paper can further the ongoing policy debate to legalize regulated Cannabis production, marketing and consumption which would help Nepalese farmers to have the option of cultivating the crop commercially, without any anxiety of being destroyed by the authority, thereby realizing a better farm income. This not only benefits farmers, the government and traders will also benefit contributing to a reduction in a huge trade gap the country is currently experiencing, expanding more revenue base for the government expenditure which otherwise is missed at the moment, and more importantly employment generation in the rural economy which currently is experiencing mass exodus in the absence of stable employment sources (Khanal et al. 2021; Pathak, et al. 2024). Such benefits, while minimizing social harm, is evidenced from the countries legalizing Cannabis production and consumption (Pathak, et al. 2024).

## Methodology

### Research location 

For this study, we selected the Tarakhola rural municipality of Baglung district in Gandaki province because of the high probability of surveying Cannabis farmers and documenting Cannabis agriculture on the basis of discussions with concerned stakeholders in Kathmandu and Pokhara, which are the largest market of Cannabis, specifically hemp textile products, in Nepal. When we compare other areas of Nepal, the western and far western mid-hills may be more suitable for Cannabis cultivation, though it is cultivated across the mid-hills of Nepal including Central and Eastern Nepal (Bhatt 1977; Shah 1997; Gersony 2003; Clarke 2007; Aryal & Adhikari 2019; Adhikari & Shiwakoti 2020; Bista 2020). The steep-sloping terraced farms, climate, elevation, traditional agricultural practices, and relative remoteness all play considerable roles in the choices that farmers make to cultivate Cannabis. Income generation in these regions primarily comes from agriculture, and many of the farmers in these regions still practice traditional forms of agriculture, which require greater manual labor and lack technical knowledge in conventional agriculture.

The Tarakhola rural municipality consists of four major areas, Amarbhumi, Argal, Hile, and Tara; additionally, Tara is divided into two wards for a total of five wards. The rural municipality was established in 2017 by merging three village development committees (VDCs), namely, Amarbhumi, Argal, and Hile, and several wards of the Tara VDC. Previously the rural municipality consisted of 36 smaller groupings called wards (Tarakhola Rural Municipality 2022), and within those wards, there were several *tole* (hamlets). The census survey of 2021 reported 2337 households in the rural municipality with a population of 10,120, which was a decline from 12,009 in 2011 (NSO 2023). Janajati (mostly Magar and Gurung) constitutes around 53% of the total population. Nearly 80% of the population in Tarakhola rural municipality are engaged in agriculture (NSO 2023).

**Fig. 1:**
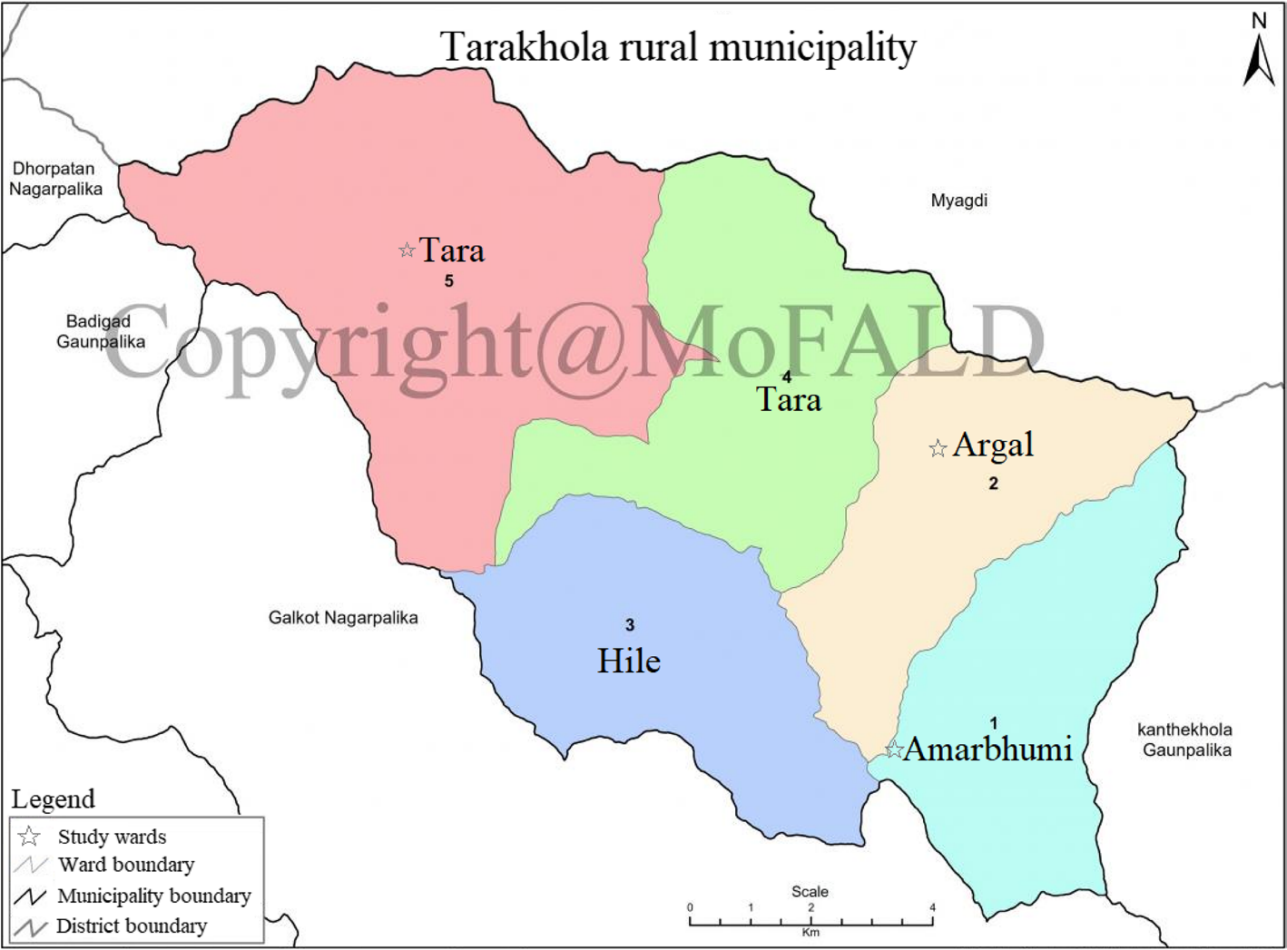
Map of Tarakhola rural municipality showing the study wards (Tarakhola Rural Municipality 2022)

Wards 1 (Amarbhumi), 2 (Argal), and 5 (Tara) were selected randomly for this study (Fig. 1). In the second step, a cluster sampling technique was used based on the hamlet information provided by the district office. The hamlets were chosen at random, and the houses within those hamlets were visited accordingly. A total of 297 households were surveyed over the course of 21 days in February to March,2019 as a follow-up survey to a series of pilot surveys conducted in 2017 and 2018. Owing to the lack of household data for the survey, the 2010 ward information from the Baglung district office was used to create our sample for the study. Table 1 shows the sample distribution based on different subsample categories.

**Table 1:** Setup of the item count technique

Control A · I`m growing corn. · I have planted coffee trees. · I planted beans. · I ate dido.	Control X · I received some income from selling sugarcane. · I planted vegetables. · I drank *chai*. · I received some income from selling citrus.
Control B · I planted potatoes. · I planted pineapple. · I planted millet. · I ate dal and rice.	Control Y · I received some income from selling cotton. · I ate some meat. · I drank hot water. · I received some income from selling mango.
**Treatment:*** I planted the cannabis plant.*	**Treatment:** *I received some income from selling the “husk” of the cannabis plant.*

## Research design

It is well documented that illicit plant cultivation occurs in many regions across the world and for a variety of reasons outside of illicit drug consumption (Ibanez & Martinsson 2013; Chouvy & Macfarlane 2018). Since Cannabis cultivation is illegal, it is assumed that estimating which farmers cultivate through self-reporting would be negatively biased due to the stigmatizing behavior of illegal activity. Surveys reporting on sensitive issues tend to be negatively biased, as survey participants are less likely to admit to being involved in illegal activities (Biemer, Jordan, Hubbard, & Wright, 2005). Our study also focused on the cultivation of Cannabis and the potential for the harvested material to be used in illicit drug production. The study utilizes the item-count technique for data analysis.

The item count technique or list experiment can be a useful method for estimating the behaviors or actions of certain populations that take part in illegal or socially sensitive activities. This technique can also estimate a percentage of the population involved without actually naming the person(s) involved. We select the key item(s) that are considered sensitive. Several other “nonkey” items were also selected and ordered in a list based on interviews with key informants from the studied population. The choice of the nonkey item needs careful consideration. It may be important to keep nonkey items thematically close to the sensitive item (Biemer, Jordan, Hubbard, & Wright, 2005). The nonkey items should have low and high prevalence so that both positive and negative responses can be maintained (Blair & Imai, Statistical analysis of list experiments, 2012).

In common applications, the list experiment estimator is weak in detecting sensitivity biases because of the bias‒variance tradeoff (Blair et al. 2020). Hence, the double-list experiment was applied to provide a clearer estimate by testing the same treatment statement with two subsamples (Droitcour, et al. 1991). The double list experiment reduces variability by half in estimates without compromising bias reduction (Miller 1984; Diaz 2023). To test the impacts of the treatments, two baseline lists are required so that the sensitive statement, as a treatment, can be presented to all respondents. The additional lists B and Y must also be correlated with A and X, respectively, thus improving the accuracy of the estimation (Glynn 2013).

In the survey, two separate baseline lists were created for each of our two treatments being tested (Table 1). Table 1 shows the control lists used in the survey, and depending on group I or II, the treatment was added to the end of each of the lists accordingly. The lists were created through discussion with agricultural experts from the district livestock and agriculture office and should accurately reflect choices made by farmers in these regions on an annual basis. For example, farmers typically drink *chai* (tea) or hot water after meals daily but do not sell cotton or sugarcane in this region, as they are not crops grown in this region. Hence, the lists have a negative within-list correlation contributing to low variance and bias caused by ceiling/floor effects and a positive between-list correlation to reduce variance (Glynn 2013).

Table 2 shows the number of farmers involved in our survey and their questionnaire types. For Subsamples A (n = 105) and B (n = 105), a set of cards was presented to each household head, and a questionnaire was followed by the ICT survey. Subsample C (n = 87) received a direct question survey about Cannabis production as well as a household questionnaire and was omitted from the ICT survey. The questions i) *“I planted the cannabis plant”* (PC) and ii) *“I received some income from selling the “husk” of the cannabis plant”* were the sensitive treatment statements denoted SS in Table 2. The sensitive statement questions were included in the direct questionnaire survey. The survey was conducted in Nepali, and the farmers were informed of the conditions of the survey and the maintenance of their privacy following the survey.

**Table 2:** Sample distribution

	Subsample I	Sub-Sample II	Subsample III
Questionnaire	A Baseline	B Baseline	Direction question
	B Baseline + SS PC	A Baseline + SS PC	
	X Baseline	Y Baseline	
	Y Baseline + SS SH	X Baseline + SS SH	
Sample Size	105	105	87

*SS* *PC* stands for “sensitive statement – I planted the cannabis plant”, *SS SH* stands for “sensitive statement—*I received some income from selling the “husk” of the cannabis plant*”

An instruction card was read to all the participants:

*We have some cards that describe many of the daily life activities that are happening here in the village. I want you to read the following statements. After reading, tell us the number of activities you are personally doing each year. You do not need to say which activities you are doing just tell us* ***HOW MANY*** *of the activities you are doing from the list.*

Depending on whether the respondent was marked with I or II cards, A_4_, B_5_ and X_4_, Y_5_ or A_5_, B_4_ and X_5_, and Y_4_ were handed to the participants. A and B were alternated through 6 households. The 7 th household would receive a direct questionnaire survey and would not take part in the item count survey, thus constituting subsample III.

The effect of a treatment with the sensitive statement “received some income from the husk” can be estimated through the Double-List Survey Equation (Tsai 2019) (Eq. 1).

1 $${\widehat{p}}_{SH}=\frac{1}{2}[\left({\overline{X} }_{{5}^{II}}-{\overline{X} }_{{4}^{I}}\right)+\left({\overline{Y} }_{{5}^{I}}{-\overline{Y} }_{{4}^{II}}\right)$$

where ${\widehat{p}}_{SH}$ is the proportion of farmers who received some income from selling the husk of the Cannabis plant, ${\overline{X} }_{{5}^{II}}$ is the mean number of statements on the 5-statement list X (including sensitive statements) counted by farmers in subsample II, ${\overline{X} }_{{4}^{I}}$ is the mean number of statements on the 4-statement list X counted by farmers in subsample I, ${\overline{Y} }_{{5}^{I}}$ is the mean number of statements on the 5-statement list Y (including sensitive statements) counted by farmers in subsample I, and ${\overline{Y} }_{{4}^{I}}$ is the mean number of statements on the 4-statement list Y counted by farmers in subsample I.

Additionally, the respondents in subsample I received the A baseline list and then received the B baseline list with the sensitive statement “I planted a cannabis plant”, whereas the respondents in subsample II received the B baseline list and then a baseline list with the same sensitive statement. The effect was estimated through Eq. 2.

2 $${\widehat{p}}_{PC}=\frac{1}{2}[\left({\overline{A} }_{{5}^{II}}-{\overline{A} }_{{4}^{I}}\right)+\left({\overline{B} }_{{5}^{I}}{-\overline{B} }_{{4}^{II}}\right)$$

where ${\widehat{p}}_{PC}$ is the proportion of farmers who planted Cannabis, ${\overline{A} }_{{5}^{II}}$ is the mean number of statements on the 5-statement list A (including sensitive statements) counted by farmers in subsample II, ${\overline{A} }_{{4}^{I}}$ is the mean number of statements on the 4-statement list A counted by farmers in subsample I, ${\overline{B} }_{{5}^{I}}$ is the mean number of statements on the 5-statement list B (including sensitive statements) counted by farmers in subsample I, and ${\overline{B} }_{{4}^{I}}$ is the mean number of statements on the 4-statement list B counted by farmers in subsample I.

The order of items on the short list (without the SS control) and the long list (with the SS treatment) were randomized to minimize order effects (Blair & Imai 2012). Similarly, three diagnostic tests were performed to check the validity of the experiment (Blair & Imai 2012; Tsai 2019). First, there are differences between the short list (control) and long list (treatment) groups in terms of important socioeconomic variables, such as age, gender, marital status, ethnicity, working in agriculture, working outside the home, landholding, and employment, to confirm the treatment randomization. The nonsignificant difference in the variables considered (Table 3) suggests that the treatment should be randomly assigned. Second, no-liar movement through the floor or ceiling effects occurred, and finally, no design effects were observed. These diagnostic tests ensure three assumptions for the list experiment if fulfilled, hence the use of a difference-in-means estimator to estimate the prevalence of ‘planting cannabis’ and ‘sold husk’ in the respondents.

**Table 3:** Distribution of the samples

Variables	IC I (n= 105)	IC II (n= 105)	*p *Value IC I vs IC II	DQ (n= 87)	*p *Value IC I vs DQ	*p *Value IC II vs DQ	IC (n= 210)	p Value IC vs DQ
Age			0.21		0.33	0.11		0.18
< 25 years old	14 (13.3)	10 (9.5)		5 (5.7)			24 (11.4)	
25–40 years old	32 (30.5)	46 (43.8)		26 (29.9)			78 (37.1)	
>40–60 years old	43 (41.0)	33 (31.4)		39 (44.8)			76 (36.2)	
> 60 years old	16 (15.2)	16 (15.2)		17 (19.5)			32 (15.2)	
Gender			0.67		0.17	0.08		0.08
Male	40 (38.1)	43 (41.0)		25 (28.7)			83 (39.5)	
Female	65 (61.9)	62 (59.0)		62 (71.3)			127 (60.5)	
Marital status			0.61		0.609	0.66		0.72
Married	96 (91.4)	97 (92.4)		77 (90.6)			193 (91.9)	
Single	8 (7.6)	8 (7.6)		8 (9.4)			16 (7.6)	
** Divorced**	1 (1.0)	-		-			1 (0.5)	
Ethnicity			0.69		0.1	0.12		0.11
Bahun	14 (13.3)	17 (16.2)		22 (25.3)			31 (14.8)	
Chhetri	10 (9.5)	6 (5.7)		9 (10.3)			16 (7.7)	
Janajati	61 (58.1)	64 (61.0)		48 (55.2)			124 (59.3)	
Dalit	20 (19.0)	18 (17.1)		8 (9.2)			38 (18.2)	
Adults Working in Agriculture	2.66 (0.13)	2.39 (0.13)	0.14	2.49 (0.13)	0.36	0.57	2.52 (0.09)	0.86
Yes	105 (100)	103 (98.0	0.16	84 (96.6)	0.06	0.50	208 (99.0)	0.13
No	0 (0)	) 2 (2.0)		3 (3.4)			2 (1.0)	
Working outside of the home	0.99 (0.12)	0.76 (0.09)	0.12	0.90 (0.12)	0.58	0.35	0.88 (0.07)	0.88
Yes	59 (56.2)	54 (51.4)	0.49	50 (57.5)	0.86	0.40	113 (53.8)	0.56
No	46 (43.8)	51 (48.6)		37 (42.5)			97 (46.2)	
Landholding (hectares)	0.66 (0.11)	0.50 (0.08)	0.24	0.59 (0.09)	0.65	0.44	0.58 (0.07)	0.90
≤ 2 hectares	93 (88.6)	98 (93.3)	0.23	82 (87.0)	0.17	0.79	191 (91.0)	0.34
> 2 hectares	12 (11.4)	7 (6.7)		5 (5.0)			19 (9.0)	
Employment			0.46		0.54	0.85		0.56
Employed	14 (13.3)	9 (8.6)		9 (10.3)			23 (21.0)	
Self-employed	87 (82.9)	90 (85.7)		72 (82.8)			177 (84.3)	
Unemployed	4 (3.8)	6 (5.7)		6 (6.9)			10 (4.8)	

Figures in parentheses indicate percentages; IC I received the control list of A/X and the treatment list of B/Y, and IC II received the control list of B/Y and the treatment list of A/X

The demographic and socioeconomic determinants of sensitive statements are estimated through a least square multivariate regression model suggested by Tsai (2019).

## Results and Discussion

### Tests of three assumptions

#### Socioeconomic status of the respondents (balance test)

Table 3 shows the descriptive statistics of the respondents under item count I (IC I), item count II (IC II), item count I and II combined (IC), and direct question (DQ), along with the differences in the socioeconomic status of the respondents among these four categories. The statistically insignificant p values for all the variables suggest that the groups be balanced and that the treatment is random, fulfilling one important assumption of the list experiment (item count techniques). Most of the respondents (72–75.2%) were aged between 25 and 60 years. Similarly, the majority of the respondents were female, which was due mainly to the outmigration of male members. Females constitute close to 55% of the total population in the study area (CBS, 2012). Approximately 92% of the respondents were married. As the study area is dominated by Janajati, more specifically Magar, the majority of the respondents belong to Janajati (mostly Magar and few Gurung), followed by Bahun/Chhetri and Dalit. Almost all households have their adult member(s) working in agriculture. On average, approximately 2.5 members are working in agriculture in the study area. More than 50% of the respondents had few members working outside of the home. This figure is close to the 55.8% of households receiving remittance in Nepal in 2011 (NSO 2024). The average landholding size of the respondents is 0.58 hectares, with a vast majority of them holding less than 2 hectares. In the case of employment, more than 80% of the respondents are self-employed, predominantly in agriculture.

#### Distribution of item counts—confirming the no-liar effect (no floor or ceiling effect)

**Fig. 2:**
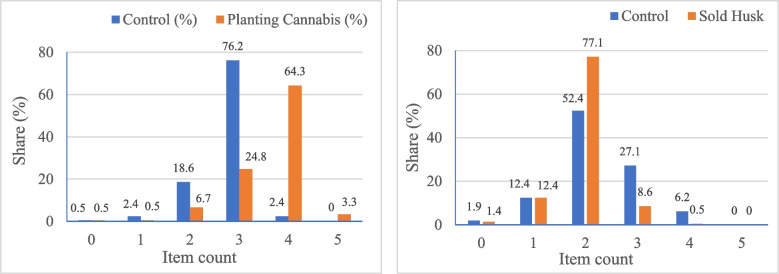
Distribution of item counts for ‘planted cannabis’ (left) and ‘sold husk’ (right)

The no-liar effect requires respondents on the long list (treatment group) to answer the sensitive statement truthfully. It is statistically not feasible to check assumptions, as respondents’ answers to the key item are unobserved by design, and their truthful answers are unknown (Tsai 2019). The distribution of item counts provides some indication of no floor or ceiling effects (Asadullah et al. 2021). The concentrations of responses in extreme cases, either 0 (on the left) and/or 4 for the control and 5 for the treatment (on the right), indicate the presence of floor and/or ceiling effects, respectively. A very small proportion of extreme cases at both ends, as depicted in Fig. 2, suggest the no-liar effect. The distribution of item counts for different groups of lists also suggests an absence of a liar effect (Annex 1).

#### No-design effect

No design effect assumes that answers to nonkey items by respondents are not affected by the inclusion of sensitive statements (key items) in the list. In the case of the list experiment for ‘planting cannabis’, a statistical test for the no-design-effect assumption is performed as proposed by Blair & Imai (2012) (Tsai 2019). The null hypothesis in the test indicates no design effect. All the nonsignificant p values in Table 4 indicate that the null hypothesis prevails, i.e., there is no design effect in the list experiment for ‘planting cannabis’. This is further validated by two hypothesis tests in the second part of the table. The first hypothesis is that none of the Pr values (R = r, S = 0) are smaller than zero, and the second hypothesis is that none of the Pr values (R = r, S = 1) are smaller than zero. The rejection of either of these hypotheses with a significant p value indicates the presence of a design effect. In this study, both the insignificant p values suggest acceptance of the null hypothesis, i.e., the design effect. Similar results prevail for both groups of list experiments (Annex 2).

**Table 4:** Joint distributions of the key and nonkey items in the list experiment for ‘planted Cannabis’

		Coefficient	Robust SE	p values
Pr(R= 0, S= 1)		0.000	0.007	0.500
Pr(R= 0, S= 0)		0.005	0.005	0.842
Pr(R= 1, S= 1)		0.019	0.013	0.924
Pr(R= 1, S= 0)		0.005	0.008	0.719
Pr(R= 2, S= 1)		0.138	0.034	1.000
Pr(R= 2, S= 0)		0.048	0.022	0.986
Pr(R= 3, S= 1)		0.652	0.034	1.000
Pr(R= 3, S= 0)		0.110	0.043	0.995
Pr(R= 4, S= 1)		0.033	0.012	0.996
Pr(R= 4, S= 0)		− 0.010	0.016	0.279
Test for design effects (with generalized moment selection)				
Ha:Pr< 0	K	Lambda	P>Lambda	#p values
Pr(R, S= 0)	1	0.343	0.279	0.558
Pr(R, S= 1)	1	0.000	0.500	1.000

#Bonferroni-adjusted p values

In the case of the list experiment for ‘sold husk’, however, none of the respondents in the list experiment responded with item count 5 on the long list, as shown in Fig. 2 (right). Hence, the study relied on the diagnostic test, where a positive value of differences between the treatment and control groups in the proportions of participants with at least one positive response indicates that the presence of a design effect is unlikely (Huber-Krum, et al. 2020). Differences between the treatment (Row 2 ‘Proportion at least’ in Table 5) and control (Row 4, ‘Proportion at least’ in Table 5) groups in the proportion of participants with at least one positive response (Row 5 in Table 5) for all ‘Number of reported items’ are positive. Thus, the presence of a design effect is unlikely for this list experiment as well. Assessments of the no-design effect for the two different lists separately also suggest that the presence of the design effect is less likely for both groups of lists (Annex 3).

**Table 5:** Response proportions by number of reported items in the list experiment for ‘sold husk’, aggregated

Rows	Source	Number of reported items						Sum
		0	1	2	3	4	5	
Row 1	List with'sold husk'	0.00	0.12	0.52	0.27	0.06	0.00	1.00
Row 2	Proportion at least	1.00	0.98	0.86	0.33	0.06	0.00	-
Row 3	List without'Sold husk'	0.01	0.12	0.77	0.09	0.00	0.00	1.00
Row 4	Proportion at least	1.00	0.98	0.86	0.09	0.01	0.00	-
Row 5	Row 2 minus Row 4	0.00	0.00	0.00	0.24	0.05	0.00	0.29

The sum of Row 5 gives the difference-in-means estimator for the prevalence of ‘sold husk’ in the study area

### Prevalence of Cannabis cultivation and husk selling

**Fig. 3:**
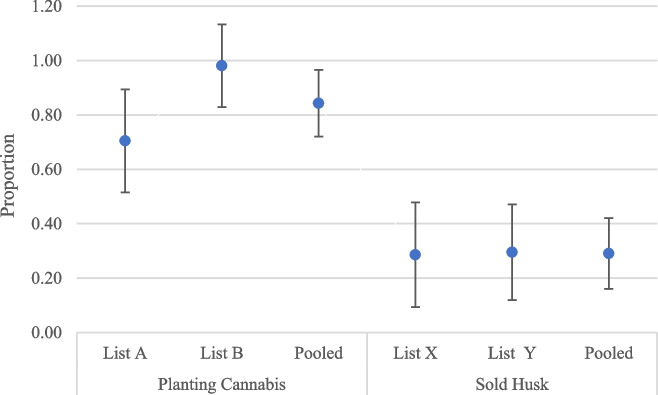
Prevalence of planting Cannabis and selling husk. Note: Vertical lines denote 95% confidence intervals

Approximately 97% of the respondents said that they cultivated Cannabis. This figure is even greater than that reported by the list experiment. The difference-in-mean estimator of the list experiment suggested that 84.3% of the respondents cultivated Cannabis (Fig. 3 and Annex 4). There was a relatively high difference in the prevalence of Cannabis cultivation estimated from List A and List B. List A suggested that 70% of the respondents cultivated Cannabis, whereas List B suggested that 98% of the respondents cultivated Cannabis in the study area (Fig. 3). This finding indicates that Cannabis cultivation is a nonsensitive behavior and remains part of the annual agricultural practice in the study area. The crop has socioeconomic and religious-cultural significance in Nepali society. Fiber and fiber-based entrepreneurship are not restricted in western Nepal, which results in the demand for Cannabis fiber (hemp fiber), further encouraging farmers to cultivate Cannabis. In addition, seeds also have important market value as food. Similarly, the crop has religious significance for Hindus, specifically lord Shiva revered by some ethnic groups as well. Hence, it is cultivated throughout Nepal even though it is not allowed by law; therefore, there is frequent reporting of a routine destruction of Cannabis plants by Nepal police in the news (Adhikari & Shiwakoti 2020; Aryal & Adhikari 2019; Bhatt D. D., 1977; Bista 2020).

**Fig. 4:**
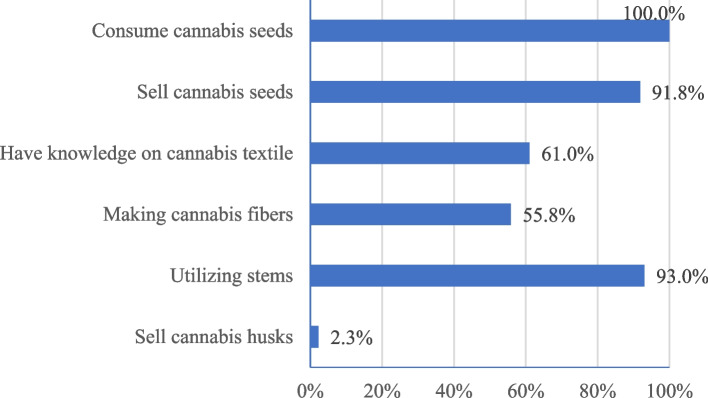
Knowledge and use of Cannabis plants

Direct questions revealed that all the respondents were consuming Cannabis seeds (Fig. 4). Most (58%) of them consumed Cannabis seeds daily as a food. Sauce (*achar*) made of Cannabis seeds, popularly known as *bhaang ko achar,* is a popular side dish. Thus, Cannabis cultivation provides people with a locally available source of protein, fiber and healthy fatty acids. Moreover, it generates income for 92% of the respondents. On average, each household produces approximately 112 kg (kg) of Cannabis seeds, with a range of 12.5 kg to 500 kg. Cannabis seeds from western Nepal are well known in different market centers, such as Kathmandu, Pokhara, Narayanghat/Bharatpur, and Malekhu, among others; the average price of Nepali Rupees (NRs) is 160 per kg, with a range of NRs of 120–320 per kg. The respondents also mentioned that soap, oil, rice, spices, sugar, and salt could be traded for their Cannabis seeds. A relatively small proportion (61%) of the respondents had knowledge of Cannabis textiles.

In addition to the main harvest of Cannabis seeds and fiber, 93% of the respondents utilized woody Cannabis stalks (stems). Cannabis stems are used as an alternative to locally sourced wood for cooking and heating. This helps ease the pressure on nearby forests for fuelwood. Some farmers also make ropes from Cannabis fibers extracted from the stem, but very little is sold and is mostly used in the household. The stems are also used for making temporary boundary walls in rural areas and environmentally friendly construction materials such as hempcrete in urban areas of Nepal.

The presence of the psychoactive compound tetrahydrocannabinol (THC), which is present mainly in the leaves and buds of Cannabis plants, has been the main reason for Cannabis cultivation in many countries around the world, including Nepal. Cannabis husks also contain tetrahydrocannabinol (THC), a psychoactive compound that is used illicitly in the study area. Asking a direct question if the respondents sell ‘hashish’ or received income from selling hashish would be offending. Hence, ‘selling husk’ is regarded as a sensitive item in this study. Receiving income from the sold husk would be considered anti-social behavior, as farmers were much less likely to reveal sold husks when asked directly. Only 2.3% of the respondents in the direct question selected ‘Yes’ for the question ‘Did you obtain extra income from selling Cannabis husk?’ (Fig. 4). In contrast, the list experiment indicates that 29.1% of the respondents were involved in selling Cannabis husk and thereby ‘*received some income from selling the cannabis “husk”.* The prevalence of selling husks established by Lists X and Y and aggregating are statistically significant (Annex 4). This finding indicates that Cannabis husk remains a source of cash income for farmers in the rural region of western Nepal, despite not being permitted by the law. The two farmers who revealed selling in the direct questionnaire revealed that farmers could gain 50,000 NRs per year from selling husks or 8000 NRs per kg, which was greater than what some farmers revealed earning per year. Importantly, not all farmers consider selling their husks for illicit purposes. Many farmers till the husk material back into the field as a “green manure” to increase the amount of organic matter in the soil, thereby improving soil fertility. Thus, along with the low external input-based production and management practices, these multiple uses of Cannabis plant parts such as seeds for food, stalk for fiber, leaves for fodder, any remaining plant parts to improve soil quality and the whole plant parts are used in ethnomedicinal practice to cure diarrhea, dysentery, cholera, cold, constipation, and snake bites (Sapkota 2008; Bhatt et al. 2021), Cannabis cultivation would be the more sustainable alternative to existing chemical intensive conventional agricultural practice with associated health risk to producers as well as consumers.

### Factors determining ‘husk selling’

As 97% of the respondents in the direct questionnaire and slightly more than 98% of the respondents in the List experiment (List B) responded that they are cultivating Cannabis, planting Cannabis does not seem to be a sensitive statement. Similarly, close to 98% of the respondents who responded to the direct question reported not selling the husk. Hence, only a list experiment for ‘husk selling’ is considered for the determinants of positive response to the sensitive statement. Separate least square multivariate regression models were run for List-X, List-Y and the pooled model. The results presented in Table 6 suggest that the age category of the respondents, their employment status, their engagement in agriculture, and their land holdings significantly affect husk selling. Older respondents (aged more than 65 years) are more engaged in husk selling than younger respondents are (≤ 40 years old). Complementing the limited cash income could be the reason for them selling husks. Compared with being employed, self-employment also has a positive significant effect on husk selling. Agriculture is the main source of employment for self-employed respondents. Hence, cultivating Cannabis along with the main crops is more convenient for them, thereby selling the husk when compared with employed respondents. Unemployed respondents mainly constitute students, who are rarely involved in the selling of agricultural products, including Cannabis husk. Hence, unemployment has a significant negative effect on the selling of Cannabis husk. Compared with not engaging in agriculture, engaging in agriculture has a positive significant effect on husk selling. The landholding activities of the respondents had a positive significant effect on husk selling. Landholdings larger than 2 hectares significantly increase the chance of husk selling.

**Table 6:** Determinants of ‘husk selling’

Variables	List-X			List-Y			Pooled		
	**Coefficient**	**RSE**	**P-va**	**Coefficient**	**RSE**	**P-va**	**Coefficient**	**RSE**	**P-va**
*Age category (Base – Youth till 40 years old)*									
Middle age 41–64 years old	− 0.291	0.22	0.19	0.278	0.19	0.13	− 0.065	0.14	0.69
Aged above 64 years old	**0.576**	**0.32**	0.07^*^	**0.342**	**0.283**	**0.23**	**0.460**	**0.22**	0.04^**^
*Gender (Base – Female)*									
Male	0.016	0.23	0.94	0.187	0.19	0.32	− 0.104	0.15	0.49
*Marital status (Base – Married)*									
Married	0.400	0.29	0.17	0.004	0.35	1.00	0.212	0.24	0.37
Divorced	-	-	-	− 0.405	0.41	0.33	0.320	0.28	0.25
*Caste/Ethnicity (Base – Bahun)*									
Chhetri	0.497	0.39	0.21	− 0.571	0.42	0.17	− 0.070	0.30	0.82
Janajati	0.270	0.30	0.37	0.276	0.33	0.41	0.253	0.22	0.26
Dalit	0.269	0.411	0.51	0.428	0.41	0.30	0.341	0.28	0.23
*Employment (Base – Employed)*									
Self-employed	**− 0.413**	**0.36**	**0.25**	**0.537**	**0.25**	0.03^**^	**0.113**	**0.21**	**0.59**
Unemployment	**− 0.793**	**0.47**	0.09^*^	**0.438**	**0.56**	**0.43**	**− 0.111**	**0.33**	**0.74**
*Member working outside from home (Base – No)*									
Yes	0.106	0.19	0.58	0.070	0.18	0.70	0.083	0.13	0.54
*Members working in agriculture (Base – No)*									
Yes	**0.036**	**0.26**	**0.89**	**-**	**-**	**-**	**1.163**	**0.25**	0.00^***^
*Land category (Base –* ≤ *2 hectares)*									
> 2 hectares	**− 0.490**	**0.30**	**0.11**	**0.493**	**0.29**	0.09^*^	**0.149**	**0.24**	**0.52**
Constant	0.102	0.45	0.82	− 0.728	0.47	0.12	− 1.392	0.38	0.00^***^
Number of observations	210			210			420		

*RSE* Robust Standard Error, *P*-va P value

^***^Significant at 1%

^**^Significant at 5%

^*^Significant at 10%

## Conclusion

The utilization of Cannabis in Nepal, as documented in this survey, appears to fall under the category of traditional wider licit use when we consider Cannabis use by farmers as a food, fiber, a source of income, medicine, and a tradeable good, especially when we consider the overall nonsensitivity to planting the crop. Despite that the cultivation of Cannabis is illegal, it remains an important option to the farmers when constrained by the external inputs’ availability for conventional cash crops like vegetables along with its socio-cultural significance, hence, it is worth taking the risk of the crop being destroyed by law-enforcing bodies. Considering its potential contribution to the livelihoods of farmers in rural Nepal and to the national economy as evidenced prior to its ban, there is an ongoing policy debate to legalize the crop in Nepal. However, since illicit use also prevails in the study areas, it is necessary to assess whether illicit use offsets traditional licit use of Cannabis, necessitating more discussion at the local, state, and national levels in Nepal. It would need to undergo the same investigation and debate as other regions around the world for its legalization. This is more critical in the case where farmers are growing this socioculturally important crop, despite its illegal status, with a high anxiety of it being destroyed by law-enforcing bodies. Rather, they preferred the crop to be legalized with some degree of regulation to ease the livelihood options of the farmers as well as explore the prospects of Cannabis products in international markets. Special consideration needs to be given to aged farmers, self-employed households, households with member(s) involved in agriculture and households with larger landholdings to reduce the illicit use of Cannabis in the study areas. Moreover, considering the strong social tie among the rural residents in Nepal, legalizing Cannabis production through group licensing would be a practical option, where farmers have a group liability to overcome any illicit use of Cannabis. An elected local government could play an important role in this regard.

This paper contributes to the lack of documentation on Cannabis, especially concerning its cultivation and illicit use, which otherwise are largely under-reported unless social desirability bias is controlled for. This work could provide a foundation for establishing a legitimate market for the Cannabis crop in Nepal considering some policy provisions to tackle its illicit use as the legitimacy of production would also pave the way for the development of a regional Cannabis industry in Nepal, scaling up the traditional means of production and using natural resources to make advances in the food, medical, and construction sectors in Nepal.

We acknowledge that the in-depth qualitative interview or case studies would provide a more holistic view of the socio-economic impacts of Cannabis cultivation, which remains the limitation of our study. Similarly, along with its social and environmental significance, it is equally important to assess the possible gains to the economy and harm to society due to the legalization to ensure the sustainability of new policies and provide sustainable alternatives to contemporary external input-intensive farming practices.

## Supplementary Information

Supplementary Material 1.

## Data Availability

Data is provided within the manuscript or supplementary information files.
