# Supplementary material for: Revealed reality of cultivation and licit/illicit use of Cannabis (Cannabis sativa L.) in the western mid-hills of Nepal: a list experiment
Source: J Cannabis Res. 2025 Apr 12;7:19. doi: 10.1186/s42238-025-00276-w (PMC11992812; doi:10.1186/s42238-025-00276-w)
Supplement: Supplementary file 1 — Supplementary Material 1. [file 42238_2025_276_MOESM1_ESM.docx]

|  |  |
| --- | --- |
|  |  |

*Annex 1. Distribution of item counts for ‘planted cannabis’ (top) and ‘sold husk’ (bottom) by different list groups*

Annex 2. Joint distributions of the key and nonkey items in lists A and B for ‘planted cannabis’

|  | List A | | | List B | | |
| --- | --- | --- | --- | --- | --- | --- |
|  | **Coefficient** | **Robust SE** | **P>z** | **Coefficient** | **Robust SE** | **P>z** |
| Pr(R=0, S=1) | -0.010 | 0.009 | 0.158 | 0.010 | 0.009 | 0.843 |
| Pr(R=0, S=0) | 0.010 | 0.009 | 0.843 | 0.000 | 0.000 | 0.500 |
| Pr(R=1, S=1) | 0.010 | 0.021 | 0.675 | 0.029 | 0.016 | 0.961 |
| Pr(R=1, S=0) | 0.019 | 0.013 | 0.923 | -0.010 | 0.009 | 0.158 |
| Pr(R=2, S=1) | 0.133 | 0.053 | 0.994 | 0.143 | 0.040 | 1.000 |
| Pr(R=2, S=0) | 0.095 | 0.036 | 0.996 | 0.000 | 0.023 | 0.500 |
| Pr(R=3, S=1) | 0.552 | 0.050 | 1.000 | 0.752 | 0.044 | 1.000 |
| Pr(R=3, S=0) | 0.171 | 0.064 | 0.996 | 0.048 | 0.055 | 0.808 |
| Pr(R=4, S=1) | 0.019 | 0.013 | 0.924 | 0.048 | 0.021 | 0.989 |
| Pr(R=4, S=0) | 0.000 | 0.019 | 0.500 | -0.019 | 0.026 | 0.235 |

Test for design effects (with generalized moment selection)

| Ha:Pr<0 | List A | | | | List B | | | |
| --- | --- | --- | --- | --- | --- | --- | --- | --- |
|  | **K** | **Lambda** | **P>Lambda** | **#P>Lambda** | **K** | **Lambda** | **P>Lambda** | **#P>Lambda** |
| Pr(R, S=0) | 1 | 0.000 | 0.500 | 1.000 | 1 | 0.000 | 0.500 | 1.000 |
| Pr(R, S=1) | 1 | 1.010 | 0.158 | 0.315 | 1 | 1.010 | 0.158 | 0.315 |

Notes # Bonferroni-adjusted p values

Annex 3. Response proportions by number of reported items in the list experiment for ‘Sold husk’ by list

| Rows | Source | Number of reported items | | | | | | Sum |
| --- | --- | --- | --- | --- | --- | --- | --- | --- |
|  |  | 0 | 1 | 2 | 3 | 4 | 5 |  |
| List X | | | | | | | | |
| Row 1 | List with 'Sold husk' | 0.03 | 0.13 | 0.50 | 0.26 | 0.08 | 0.00 | 1.00 |
| Row 2 | Proportion at least | 1.00 | 0.97 | 0.84 | 0.33 | 0.08 | 0.00 | - |
| Row 3 | List without 'Sold husk' | 0.00 | 0.19 | 0.69 | 0.12 | 0.00 | 0.00 | 1.00 |
| Row 4 | Proportion at least | 1.00 | 1.00 | 0.81 | 0.12 | 0.00 | 0.00 | - |
| Row 5 | Row 2 minus Row 4 | 0.00 | -0.03 | 0.03 | 0.21 | 0.08 | 0.00 | 0.29 |
| List Y |  |  |  |  |  |  |  |  |
| Row 1 | List with 'Sold husk' | 0.01 | 0.11 | 0.54 | 0.29 | 0.05 | 0.00 | 1.00 |
| Row 2 | Proportion at least | 1.00 | 0.99 | 0.88 | 0.33 | 0.05 | 0.00 | - |
| Row 3 | List without 'Sold husk' | 0.03 | 0.06 | 0.86 | 0.05 | 0.01 | 0.00 | 1.00 |
| Row 4 | Proportion at least | 1.00 | 0.97 | 0.91 | 0.06 | 0.01 | 0.00 | - |
| Row 5 | Row 2 minus Row 4 | 0.00 | 0.02 | -0.04 | 0.28 | 0.04 | 0.00 | 0.30 |

Note: The sum of Row 5 gives the difference-in-means estimator for the prevalence of ‘Sold husk’ in the study area.

Annex 4. Least square estimators of the prevalence of sensitive statements

| List experiment type | Delta constant | | | Gamma constant | | |
| --- | --- | --- | --- | --- | --- | --- |
|  | Coef. | Robust SE | p value | Coef. | Robust SE | p value |
| Planting cannabis | | | | | | |
| List A | 0.7048 | 0.0954 | 0.000^***^ | 2.733 | 0.0526 | 0.000^***^ |
| List B | 0.9810 | 0.0764 | 0.000^***^ | 2.819 | 0.0535 | 0.000^***^ |
| A & B aggregated | 0.8429 | 0.0625 | 0.000^***^ | 2.776 | 0.0376 | 0.000^***^ |
| Sold husk | | | | | | |
| List X | 0.2857 | 0.1010 | 0.005^***^ | 1.933 | 0.0543 | 0.000^***^ |
| List Y | 0.2952 | 0.0885 | 0.001^***^ | 1.952 | 0.0493 | 0.000^***^ |
| X & Y aggregated | 0.2905 | 0.0671 | 0.000^***^ | 1.943 | 0.0367 | 0.000^***^ |

Note: Coef. – Coefficient; SE – standard error; ^***^significant at the 1% level. The delta constant is the central focus reflecting a proportion of the prevalence of planting cannabis and selling husk.
